# Results of sole postoperative interstitial, high-dose-rate brachytherapy of T1–2 tongue tumours

**DOI:** 10.1007/s00066-022-01901-w

**Published:** 2022-02-14

**Authors:** Zoltán Takácsi-Nagy, Örs Ferenczi, Tibor Major, Hironori Akiyama, Georgina Fröhlich, Ferenc Oberna, Mónika Révész, Márton Poósz, Csaba Polgár

**Affiliations:** 1 Centre of Radiotherapy, National Institute of Oncology, Ráth György St. 7–9, 1122 Budapest, Hungary; 2 Department of Oncology, Semmelweis University, Budapest, Hungary; 3 Department of Oral Radiology, Osaka Dental University, Osaka, Japan; 4 Faculty of Science, Eötvös Loránd University, Budapest, Hungary; 5 Multidisciplinary Centre of Head and Neck Tumours, National Institute of Oncology, Budapest, Hungary

**Keywords:** Tongue neoplasms, High-dose-rate, Postoperative treatment, Brachytherapy, Survival

## Abstract

**Purpose:**

To describe the results of treating tongue cancer patients with single postoperative interstitial, high-dose-rate (HDR) brachytherapy (BT) after resection.

**Methods:**

Between January 1998 and April 2019, 45 patients with squamous cell histology, stage T1–2N0–1M0 tongue tumours were treated by surgery followed by a single HDR BT in case of negative prognostic factors (close or positive surgical margin, lymphovascular and/or perineural invasion). The average dose was 29 Gy (range: 10–45 Gy) and rigid metal needles were used in 11 (24%) and flexible plastic catheters in 34 cases (76%). Survival parameters, toxicities and the prognostic factors influencing survival were analysed.

**Results:**

During a mean follow-up of 103 months (range: 16–260 months) for surviving patients, the 10-year local and regional control (LC, RC), overall survival (OS), and disease-specific survival (DSS) probabilities were 85, 73, 34 and 63%, respectively. The incidence of local grade 1, 2 and 3 mucositis was 23, 73 and 4%, respectively. As a serious (grade 4), late side effect, soft tissue necrosis developed in 3 cases (7%). In a univariate analysis, there was a significant correlation between lymphovascular invasion and RC (*p* = 0.0118) as well as cervical recurrence and DSS (*p* < 0.0001).

**Conclusion:**

Sole postoperative HDR brachytherapy can be an effective method in case of negative prognostic factors in the treatment of early, resectable tongue tumours. Comparing the results of patients treated with postoperative BT to those who were managed with surgery or BT alone known from the literature, a slightly more favourable LC can be achieved with the combination therapy, demonstrating the potential compensating effect of BT on adverse prognostic factors, while the developing severe, grade 4 toxicity rate remains low.

## Introduction

Oral tumours are the sixth most common malignancies in the world, accounting for 4% of all tumours [1]. According to the literature, about 48% of carcinomas affecting this region originate from the tongue [2, 3]. The lesions most often occur on the lateral and ventral parts of the tongue. These tumours are primarily treated by surgery. In case of minor lesions excision or partial glossectomy, laser surgery and radiotherapy (RT) are also possible. In more extended neoplasms, hemi- and total glossectomy with reconstruction and combined treatment (RT with or without chemotherapy) are indicated [3–7]. In T1–2, < 3 cm tumours, interstitial brachytherapy (BT) can be used instead of surgery with the same results [7–10]. However, if surgery is carried out on these smaller tumours, histology may require additional local postoperative treatment, for which BT is an excellent modality due to its favourable radiophysical properties. In cases reported in the literature, most patients were treated with a low-dose-rate (LDR) method [11–13].

In the current retrospective analysis, we examined the role of postoperative sole interstitial BT in tongue cancer using a high-dose-rate (HDR) technique, comparing our findings with results reported in the literature, and also studying prognostic factors influencing survival.

## Methods

Between January 1998 and April 2019, 45 patients with stage T1–2N0–1M0 (UICC [Union for International Cancer Control], TNM 7th Edition) [14] histologically confirmed squamous cell carcinoma were treated with postoperative sole interstitial BT. In all cases the radiation treatment was decided on by the tumour board of our institute on the basis of the findings of histological and imaging examinations. The attending member of our medical team informed the patient about interstitial BT, which is an accepted therapeutic method at our institute, allowing the patient to choose between external irradiation and BT. If the patient chose the latter, this was confirmed by the patient’s consent and signature. All procedures were carried out in compliance with the Declaration of Helsinki and conformed to the ethical standards of human experimentation in Hungary. Patient and tumour characteristics are shown in Table 1.

**Table 1:** Patient and tumour characteristics

Characteristic	Number of cases (%)
Mean age (year)	58.9 (range: 26–77)
*Sex*	
Female	14 (31)
Male	31 (69)
*Histology*	
Squamous cell carcinoma	45 (100)
*Side*	
Right	23 (51)
Left	22 (49)
*Differentiation*	
Grade I	21 (47)
Grade II	24 (53)
*Tumour size*	
T1	22 (49)
T2	23 (51)
*Lymph node status*	
N0	43 (96)
N1	2 (4)
*UICC stage*	
I	21 (47)
II	22 (49)
III	2 (4)
*Neck dissection*	
Yes	28 (62)
No	17 (38)
*Lymphovascular invasion*	
Yes	6 (13)
No	39 (87)
*Perineural invasion*	
Yes	8 (18)
No	37 (82)
*Tumour thickness*	
≥ 5 mm	25 (56)
< 5 mm	20 (44)
*Surgical margin*	
≥ 5 mm	2 (4)
< 5; > 2 mm	4 (9)
≤ 2; > 0 mm	29 (65)
R1	10 (22)

Exclusive primary tumour removal via surgery was performed in 17 patients (38%) with favourable histology (superficial tumours where the depth of invasion was < 5 mm, lack of lymphovascular, perineural invasion) and negative (N0) cervical status (*n* = 14), as well as in poorer general condition (*n* = 3), while out of the other 28 patients (62%), 27 (96%) had unilateral and 1 (4%) bilateral (ipsilateral I–IV, contralateral I–III) neck dissection too. Excision was applied in 11 (24%) and partial glossectomy in 34 (76%) cases. According to our institutional protocol, level I–III dissection was performed for N0 and level I–IV for N1 status. The mean time between interstitial BT (implantation) and surgery was 39 days (range: 31–65 days). Flexible plastic catheters (mean 5; range: 2–10) were applied in 34 (76%) and rigid metal needles (mean 2; range: 2–3) in 11 (24%) patients. The implantation was performed in an operating theatre under general anaesthesia, from submental approach. The plastic catheters were inserted through metal trocars which were removed after insertion. The catheters were fixed to submental skin and to the surface of the tongue with plastic buttons. Separation between needles/catheters ranged from 10–15 mm, and the source step size was 2.5 mm. Target volume was defined using information from preoperative imaging, surgical and histological reports, palpation of the tumour bed before/during implantation and since the year of 2000 computed tomography (CT) images taken for planning.

**Fig. 1:**
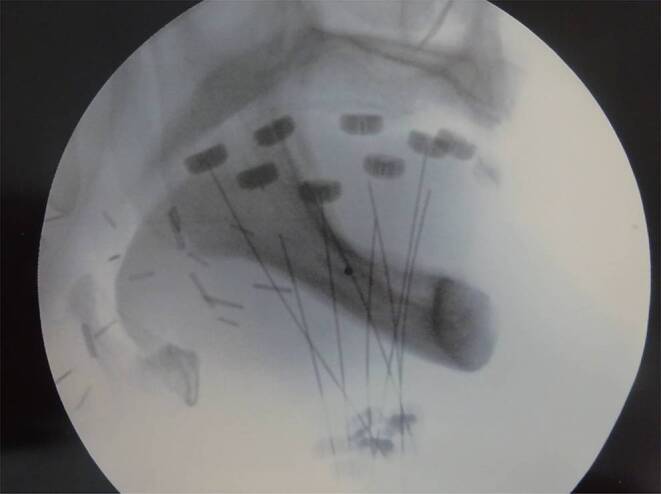
Lateral X‑ray of 8 implanted flexible plastic catheters with markers and fixing plastic buttons

**Fig. 2:**
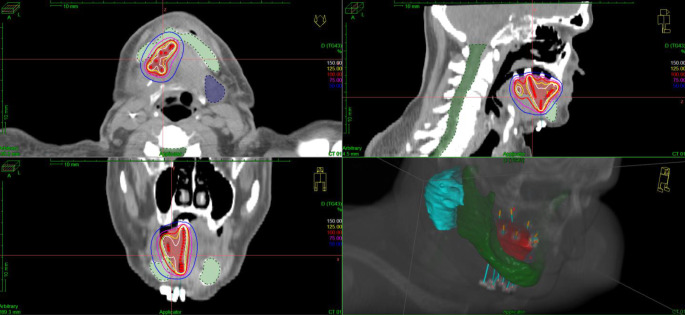
Dose distribution in relation to planning target volume (PTV, *red volume*) in axial, coronal and sagittal views, and three-dimensional computed tomography (3D-CT) reconstruction of the implanted catheters (*blue lines*) and patient anatomy on bottom right

For the first 14 patients (31%) treatment planning was based on X‑ray images taken at two different angles (Fig. 1). On the radiographs the active length of the needles/catheters was marked, and their position was digitised into the 3‑dimensional (3D) treatment planning system. The reference dose points used for dose prescription were related to the needles/catheters. They were placed at a 0.5–1.2 cm distance from the implants towards the surface of the target volume. The dose was prescribed to the mean dose in the reference points. As in 2000 CT imaging was introduced in the treatment planning, the next 31 patients (69%) were treated with CT-based planning. The planning target volume (PTV), which is equal to clinical target volume (CTV) was determined using information obtained from presurgery images, surgical and histological reports and palpation of the tumour bed. The PTV included the tumour bed with a 5 mm isotropic margin. To position the needles/catheters the rules of the Paris system were followed, but the dosimetry was based on the 3D target volume (conformal dose planning method). The reference dose points were placed on the surface of the PTV, and dose optimisation on the dose points and geometry was performed, then the dose was normalised and prescribed to the mean dose in reference points (100% isodose; Fig. 2). The number and the distance between needles/catheters were determined depending on the size of the target volume. The PTV and organs at risk were outlined on CT slices, and the source dwell positions were activated inside the PTV. A dental shield was not used during treatment.

Irradiation was performed using a Nucletron-microSelectron (Nucletron, Veenendaal, The Netherlands) HDR afterloader applying an ^192^Ir isotope with 370 GBq (10 Ci) initial source activity. For planning, first we used the Nucletron-Plato and later the Oncentra Brachy planning system (Elekta Brachytherapy, Veenendaal, The Netherlands).

The mean prescribed dose was 29 Gy (range: 10–45 Gy) corresponding to the mean 36.7 Gy (range: 16.7–48.8 Gy) equivalent dose in 2 Gy fraction (EQD2) (α/β = 10 Gy) and 47.3 Gy (range: 26–63.5 Gy) EQD2 (α/β = 3 Gy) doses (Table 2).

**Table 2:** Implantation techniques and fractionation schemes of high-dose-rate brachytherapy (HDR BT) of tongue tumours

**Technique**	**Number of patients (%)**			
Plastic catheter	34 (76)			
Rigid needle	11 (24)			
**Fractionation schemes**				
*Number of Fx*	*Dose/Fx (Gy)*	*Cases (%)*	*EQD2 (α/β* *=* *3* *Gy) (Gy)*	*EQD2 (α/β* *=* *10* *Gy) (Gy)*
1	10	2 (4.4)	26.0	16.7
1	12	12 (26.7)	36.0	22.0
1	14	2 (4.4)	47.6	28.0
6	4.0	1 (2.2)	33.6	28.0
6	5.4	1 (2.2)	54.4	41.6
6	5.5	1 (2.2)	56.1	42.6
7	4.0	1 (2.2)	39.2	32.7
7	4.2	2 (4.4)	42.3	34.8
7	4.8	1 (2.2)	52.4	41.4
7	5.0	7 (15.7)	59.7	46.1
7	5.4	1 (2.2)	63.5	48.5
8	4.0	1 (2.2)	44.8	37.3
15	3.0	13 (29.0)	54.0	48.8

*Fx* fraction

Of the 45 patients, 11 (24%) received 10–14 Gy in a single fraction using rigid metal needles. After the year of 2000 we started using multiple fractions—with the exception of five patients (1 × 10–14 Gy)—with non-loop plastic catheters, and a mean total dose of 38.3 Gy (range: 24–45 Gy) was given in 6–15 fractions (mean 11). The fraction doses were 3–5.5 Gy (mean 4 Gy) and were delivered twice daily, at least 6 h apart. From 2014 we standardised the fractionation and the total dose as follows: 15 × 3 Gy (total 45 Gy) was given, and 29% of the patients (*n* = 13) were treated by this fractionation scheme.

According to our institutional protocol, 8–10 weeks after BT control CT or magnetic resonance imaging (MRI) and physical examination were carried out during the first follow-up visit. Thereafter, every 3 months physical examination, and every 6 months in the first 2 years CT or MRI examination was performed. Furthermore, chest X‑ray and laboratory tests were made annually. The survival time was determined from the last fraction of the HDR BT. Acute and late side effects were classified according to the Radiation Therapy Oncology Group (RTOG)/European Organization for Research and Treatment of Cancer (EORTC) recommendations [15].

For 31 patients treated with CT-based planning, the mean V (PTV), V100 (percentage of the PTV receiving 100% of the prescribed dose), V150 (percentage of the PTV receiving 150% of the prescribed dose), V_x_ (volume enclosed by the surface of the x% dose), D_x_ (the dose that covers x% of the PTV), DNR (dose non-uniformity ratio: V_150_/V_100_), and the COIN (conformal index) were calculated [16, 17].

For statistical analysis the Solo software package (Department of Biometrics, University of California, Los Angeles, CA, USA) was used. The probability of survival was calculated using the Kaplan–Meier method [18]. Survival differences were compared using the log-rank test. Possible prognostic factors for local and regional control (LC, RC), overall survival (OS), and disease-specific survival (DSS) were analysed in a Cox regression model [19]. A *p*-value ≤ 0.05 was considered to represent statistical significance.

## Results

**Fig. 3:**
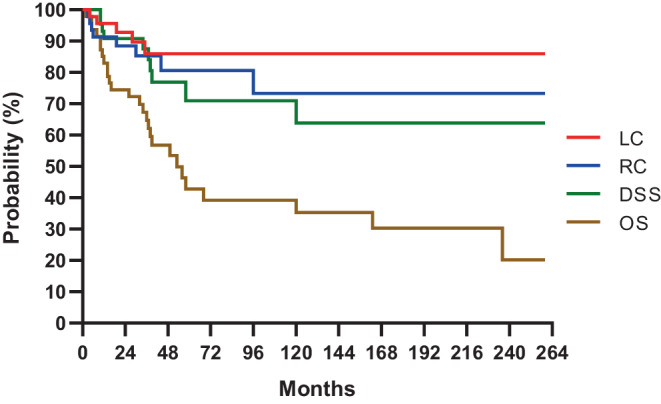
Local control (LC), regional control (RC), disease-specific survival (DSS) and overall survival (OS) for patients with T1–2 tongue tumours treated with sole postoperative interstitial, high-dose-rate brachytherapy

The mean follow-up time for surviving patients was 103 months (range: 16–260 months). No patients were lost during this period. In 10 patients (22%), there were local and/or regional recurrences (2 local [4%], 5 regional [11%] and 3 locoregional [7%]) and in 1 case (2%) distant lung metastasis occurred. Salvage treatments included surgery + external beam RT in 2 (4%), external beam RT in 1 (2%), chemotherapy in 4 (9%) and chemotherapy + external beam RT in 1 (2%) patients. Three patients (7%) received supportive treatment alone due to their poor general condition. Later on further progression occurred in these patients, so except for 1 patient (who was salvaged successfully with neck dissection and regional irradiation), 10 patients (22%) died of the primary disease, 5 (11%) due to a second primary tumour (4 lung and 1 hypopharynx) and 12 (27%) of intercurrent disease. The 5 and 10-year probabilities of LC, RC, OS, and DSS were 85% and 85% (T1 88%, T2 84%), 80% and 73% (T1 95%, T2 59%), 42% and 34% (T1 36%, T2 33%) and 70% and 63% (T1 84%, T2 51%), respectively (Fig. 3).

Univariate analysis of prognostic factors confirmed the significant effect of lymphovascular invasion on 10-year RC (77% /with negative/ vs. 40% /with positive/, *p* = 0.0118). In case of neck recurrence (*n* = 8) the histology of the primary tumour showed lymphovascular invasion in 3 (38%) patients. These 3 patients had N0 status and underwent elective neck surgery. Of the 17 patients who were treated without elective neck dissection, regional recurrence developed in 2 cases (12%), but they had no lymphovascular invasion. Cervical recurrence (no/yes) had a significant negative impact on the 10-year DSS (88% [no] vs. 0% [yes], *p* < 0.0001).

Age, gender, perineural invasion, grading, total BT dose (cut off EQD2 (α/β = 10 Gy) 36.7 Gy [mean]), number of fractions (one vs. multiple), surgical margin (positive, ≤ 2 mm, > 2 mm), tumour thickness (< 5 mm, ≥ 5 mm), neck dissection (yes or no), the time between surgery and HDR BT did not affect the survival parameters.

BT caused local grade 1, 2 and 3 mucositis in 10 (23%), 33 (73%) and 2 (4%) patients, respectively. Bacterial infections occurred in 5 (11%) and mycotic infections in 9 (20%) patients; however, all responded to antibiotic and/or antimycotic therapy. Severe (grade 4) adverse effects as soft tissue necrosis (SN) occurred in 3 cases (7%) 2–6 months (mean 4 months) after BT, but the patients recovered with conservative management. In these patients EQD2 (α/β = 3 Gy) was ≥ 59.7 Gy (7 × 5 Gy [*n* = 2], 7 × 5.4 Gy [*n* = 1]; Table 2). The mean length of time for the necrosis to heal was 4 months (range: 3–5 months). No patient needed percutaneous endoscopic gastrostomy for nutrition. Osteoradionecrosis (ON) did not occur in any of the cases. The mean D2 cm^3^ of the mandible was 1.9 Gy (57%; range: 0.2–6.1 Gy, 6.7–203.3%) which resulted in a 28 Gy (range: 1.9–166.5 Gy) EQD2 dose totally. Long-term toxicities such as xerostomia, swallowing difficulties, neck fibrosis did not occur (except in the operated neck).

The mean V (PTV) was 14.2 cm^3^ (range: 5.2–33.6 cm^3^), V100 92.8% (range: 87.9–99.4%), V150 45.6% (range: 32.3–84.9%), V_85_ 22.5 cm^3^ (range: 8.1–46.7), V_100_ 18.3 cm^3^ (range: 6.5–38.1 cm^3^), V_120_ 13.2 cm^3^ (range: 4.5–28.3 cm^3^), V_150_ 7.5 cm^3^ (range: 3.1–13.7 cm^3^), D90 105.5% (range: 90.8–138.4%), D98 88.4% (76.3–106.1%), DNR 0.42 (range: 0.32–0.55) and COIN 0.66 (range: 0.47–0.79).

## Discussion

Smaller T1–2 tumours of the oral cavity and thus of the tongue can be successfully treated with surgery or RT, with the same effectiveness. With surgery alone 63–89% LC, 75–85.6% DSS and 71–80.8% OS have been reported in the literature [20–24]. In more advanced tumours, surgery has priority and is followed by adjuvant treatment. In a study of 170 cases in which 105 patients received definitive RT ± cervical dissection and 65 underwent surgery ± postoperative RT, the 2‑year results of LC in T1–2 status were the same (76% vs. 76%); however, in T3 and in T4 cases the surgical arm showed more favourable results (82% vs. 45% and 67% vs. 0%) [23].

Due to its more advantageous radiophysical characteristic, in the definitive treatment of early tongue cancers, BT is widely used and reported in the literature. In the major studies about the BT of tongue cancers (T1–4) 70–75 Gy was given with LDR, 11 × 4 Gy or 10 × 5–6 Gy with HDR and 50–64 Gy with pulsed dose rate (PDR) technique resulting in 67–94% LTC, 47–88.7% OS with 1.4–19.8% SN and 4–12% ON occurrence [25–33].

If surgery is the chosen modality for oral T1–2 cancers, postoperative RT is indicated to reduce the risk of local recurrence in the presence of unfavourable histological parameters such as positive or close (< 5 mm) surgical margin as well as lymphovascular and perineural invasion [5, 9, 34]. Unfortunately, there are almost no reports about the results of postoperative external irradiation of these early tumours. Shim et al. [21] published data for T1–2 tongue tumours. Local recurrence occurred in 0% (0/13) with percutaneous RT and in 18% (8/44) without it. In contrast to external RT, interstitial BT is much more applicable for supplying such a small volume. However, a relatively small number of reports in the literature deal with exclusive postoperative BT in such cases (Table 3).

**Table 3:** Clinical results of sole postoperative brachytherapy in oral tongue cancer

Author Year	*n*	T status	Dose rate	Dose (Gy)	LC (%) (5 year)	RC (%) (5 year)	DSS (%) (5 year)	OS (%) (5 year)	Toxicity (grade 4) (%)
Ange 1975 [11]	17	T1–2	LDR (^226^Ra, ^198^Au)	55–60	100	88	94	82	18 (ON)
Lapeyre 2000 [12]	19	T1–2	LDR (^192^Ir)	60	95	89	NR	NR	16 (ON, SN)
Goineau 2015 [13]	112	T1–2	LDR (^192^Ir)	50–55	76	NR	67	56	22 (SN)
Strnad 2005 [33]	50	T1–2	PDR (^192^Ir)	50–64	78^a^	NR	NR	67^a^	9.7 (SN), 7.2 (ON)
Petera 2015 [35]	29	T1–3^b^	HDR (^192^Ir)	18 × 3	85	69	76	73	7 (ON), 3 (SN)
Potharaju 2018 [26]	26	T1–2	HDR (^192^Ir)	10 × 4^c^	100	96	92	92	0 (SN, ON)
Current study	45	T1–2	HDR (^192^Ir)	29^d^	85	80	70	42	7 (SN)

*n* number of patients, *T* tumour, *LC* local control, *RC* regional control, *DSS* disease-specific survival, *OS* overall survival, *LDR* low-dose-rate, *PDR* pulsed dose rate, *HDR* high-dose-rate, *NR* not reported, *ON* osteonecrosis, *SN* soft tissue necrosis

^a^LC and OS were given for 103 patients (T1–4, 87% T1–2) treated with postoperative brachytherapy ± external radiotherapy, or in 19 patients without surgery (definitive BT)

^b^only 1 patient with T3 status

^c^perioperative BT

^d^mean dose

In the current study the TNM 7th edition [14] was used for tumour evaluation. We assume that the same system or the previous TNM classifications were used in the cited publications (Table 3) because either they were published before 2017 or it is mentioned in one of the referenced articles [26]. In our patients, the 5‑year LC, RC, OS and DSS with sole postoperative HDR BT in early T1–2 tongue tumours were 85, 80, 42 and 70%, respectively (Fig. 3). As a grade 4 side effect, SN developed in 7%. In the relevant studies on this topic using LDR, HDR or PDR methods, the 5‑year LC, RC, DSS and OS were 76–100, 69–96, 67–94 and 56–92%, respectively with a rate of 0–22% SN and 0–18% ON (Table 3). Comparing the LC of patients treated with postoperative BT (76–100%) with those managed with surgery (63–89%) or BT alone (67–94%) known from the literature, a slightly more favourable LC has been achieved with the combination therapy. Based on the comparison of the results reported on in the literature, there seems to be no difference depending on the technique (LDR or HDR).

In the study of Strnad et al. [33], 103 patients (T1–4, 87% T1–2) were treated with a dose of 50–64 Gy PDR (^192^Ir) BT. Nineteen patients received BT alone, while 84 were resected (73% R0) with or without cervical dissection followed by postoperative BT ± external irradiation. The 5‑year LC was 78% and the OS was 67% with 9.7% and 7.2% SN and ON, respectively. In another study, perioperative (catheters implanted during tumour extirpation) HDR BT with 10 × 4 Gy was performed in T1–2N0 (*n* = 26). The authors reported 100% LC and 92.3% OS (5-year), without the development of late toxicity [26].

In a retrospective study, Cheng et al. [24] also draw attention to the need of postoperative RT in case of unfavourable prognostic factors. In their series 199 patients (T1–2N0) who underwent surgery alone, the locoregional relapse rate was 27%. In our study, the same parameter with exclusive postoperative BT was 5% lower at 22%. The results of our work with the analysis of the highest number (*n* = 45) of patients treated with HDR BT so far, do not differ from those of the above-mentioned studies, except for OS, which was found to be lower in our series: 27% of our cases died of intercurrent diseases, 11% of them died of a second primary tumour (mainly lung cancer). The explanation of the poorer results in these patients is that excessive alcohol consumption and smoking caused comorbidities or other cancers, which together contributed to their early death.

In T1–2 tumours, the need for elective care of the N0 neck to treat subclinical cervical metastases—by dissection or irradiation—is largely determined by the depth of tumour invasion as a prognostic factor. Seventeen (38%) of the patients did not receive elective dissection because the depth of tumour invasion was < 5 mm with N0 cervical status. Only two of them (4% of all patients) developed cervical metastases later. Fukano et al. [36] drew attention to the importance of the depth of tumour invasion. They found subclinical cervical metastases in tongue tumours in 64.7% at >5 mm depth invasion and in 5.9% below this value. Potharaju et al. [26] detected RC rates of 79.7 and 0% (*p* = 0.0001) after 6 years in tongue cancer patients without neck dissection at ≤ 5 or > 5 mm invasion, respectively. Shim et al. [21] found that the depth of invasion (cut-off 5 mm) was a significant prognostic factor affecting DSS (66 vs. 92%, *p* = 0.013).

Other authors also emphasize the prognostic role of perineural and lymphovascular invasion. Regarding perineural invasion, regional metastasis occurred in 71% (with positive) vs. 36% (with negative), while for lymphovascular invasion in 88% (with positive) vs. 38% (with negative) during the follow-up time in the case of tongue and floor of mouth localisation and N0 neck [37]. Goineau et al. [13] mentioned less favourable DSS with lymphovascular invasion (*p* = 0.044). In our analysis, a significant correlation was found between lymphovascular invasion and cervical recurrence. The 10-year RC was 77% (with negative) vs. 40% (with positive) (*p* = 0.0118). Based on this result, possible irradiation of the neck in case of lymphovascular invasion may be considered even after elective neck dissection to reduce cervical recurrences. However, in a retrospective analysis of 571 surgically treated oral cancer patients, Adel et al. [38] found that lymphovascular spread might not necessarily be an indicator for postoperative adjuvant radiotherapy.

The importance of an appropriate surgical margin is highlighted by Al-Rajhi et al. [22] who reviewed the data of 85 T1–2N0 tongue cancer patients treated exclusively with surgery and proved a significantly better DSS (*p* = 0.005) and relapse-free survival (*p* = 0.0002) at > 5 mm surgical margin.

In our study, age, gender, perineural invasion, tumour grade, dose (cut off EQD2 (α/β = 10 Gy) 36.7 Gy [mean]), fraction number (one or multiple), surgical margin (positive, ≤ 2 mm, > 2 mm), tumour thickness (< 5 mm, ≥ 5 mm), neck dissection (yes or no) and the time interval between surgery and BT did not influence survival parameters.

Soft tissue necrosis was observed in 3 patients (7%) whose EQD2 (3) was ≥ 59.7 Gy. In these cases, the dose was ≥ 5 Gy. The recommendation of GEC-ESTRO (Group of European Curie Therapy [GEC] and the European Society for Radiotherapy and Oncology [ESTRO]) for HDR head and neck BT does not suggest a dose/fraction > 4 Gy in order to reduce tissue injury [9]. A 60 Gy EQD2 (α/β = 3 Gy) or higher dose is also questionable.

It is a limitation of the present study that it is a retrospective analysis and that the doses and applied number of fractions were heterogeneous, which was largely due to the inconvenience caused by rigid metal needles used previously, resulting in a limited implantation potential. Also lack of our sufficient HDR-BT-related experience was a drawback. During the study period in our practice, there was a trend regarding fractionation and dose prescription towards a radiobiologically more favourable fractionation scheme. Initially, for the reasons described above, single fractions were delivered with a higher dose, but later the number of fractions were gradually increased, and simultaneously the dose per fraction was decreased along with escalating the total dose. However, our results did not confirm the negative effect of these above-mentioned factors on the survival parameters. Since 2014, 15 × 3 Gy has been used in sole postoperative BT in accordance with the international recommendations [9, 10] and up to now it has been well tolerated by the patients without grade 4 toxicity.

## Conclusion

This retrospective study with 45 patients is the most extensive analysis to date, investigating the role of high-dose-rate (HDR) brachytherapy (BT)in the sole postoperative treatment for tongue cancer. Based on the results, in case of negative prognostic factors sole postoperative HDR BT can be an effective method in the treatment of early tongue tumours. Comparing the results of patients treated with postoperative BT with those managed with surgery or BT alone known from the literature, a slightly more favourable local control has been achieved with the combination therapy, demonstrating the potential compensating effect of BT on adverse prognostic factors and suggesting the advantage of postoperative BT over definitive BT, while the severe, grade 4 toxicity rate has remained low. Further prospective studies are needed to standardise dose fractionation schedules for HDR BT of oral cavity (tongue) tumours and to compare the results of postoperative BT with those of BT alone in tongue cancer, and HDR BT with low-dose-rate BT.
